# Enhancement of protein production in *Aspergillus niger* by engineering the antioxidant defense metabolism

**DOI:** 10.1186/s13068-024-02542-0

**Published:** 2024-06-29

**Authors:** Xin Chen, Baoxiang Pan, Leyi Yu, Bin Wang, Li Pan

**Affiliations:** School of Biology and Biological Engineering, South China University of Technology, Guangzhou Higher Education Mega Center, Guangzhou, 510006 China

**Keywords:** *Aspergillus niger*, Oxidative stress, GSH, Protein production, *Glr1*

## Abstract

**Background:**

Research on protein production holds significant importance in the advancement of food technology, agriculture, pharmaceuticals, and bioenergy. *Aspergillus niger* stands out as an ideal microbial cell factory for the production of food-grade proteins, owing to its robust protein secretion capacity and excellent safety profile. However, the extensive oxidative folding of proteins within the endoplasmic reticulum (ER) triggers ER stress, consequently leading to protein misfolding reactions. This stressful phenomenon results in the accelerated generation of reactive oxygen species (ROS), thereby inducing oxidative stress. The accumulation of ROS can adversely affect intracellular DNA, proteins, and lipids.

**Result:**

In this study, we enhanced the detoxification of ROS in *A. niger* (SH-1) by integrating multiple modules, including the NADPH regeneration engineering module, the glutaredoxin system, the GSH synthesis engineering module, and the transcription factor module. We assessed the intracellular ROS levels, growth under stress conditions, protein production levels, and intracellular GSH content. Our findings revealed that the overexpression of *Glr1* in the glutaredoxin system exhibited significant efficacy across various parameters. Specifically, it reduced the intracellular ROS levels in *A. niger* by 50%, boosted glucoamylase enzyme activity by 243%, and increased total protein secretion by 88%.

**Conclusion:**

The results indicate that moderate modulation of intracellular redox conditions can enhance overall protein output. In conclusion, we present a strategy for augmenting protein production in *A. niger* and propose a potential approach for optimizing microbial protein production system.

**Supplementary Information:**

The online version contains supplementary material available at 10.1186/s13068-024-02542-0.

## Background

*Aspergillus niger* is distinguished by its notable safety profile, with numerous derivatives being recognized as Generally Regarded As Safe (GRAS) by the U.S. Food and Drug Administration [1, 2]. This microorganism exhibits an exceptional capacity for protein secretion, facilitating the synthesis of a broad spectrum of industrial enzymes, including glucoamylase, amylase, glucose oxidase, pectinase, peroxidase, phytase, xylanase, and cellulase [3]. Furthermore, it can grow in low-cost biomass sources, considerably lowering industrial production costs. These extraordinary characteristics establish *A. niger* as an ideal platform for the production of edible enzymes and food-grade proteins, emphasizing its critical significance in biotechnological applications.

*A. niger*, being an obligate aerobic organism, encounters the formation of superoxide anion radicals under aerobic conditions due to the incomplete reduction of molecular oxygen. These radicals can subsequently transform into hydrogen peroxide or other ROS, which possess a higher reactivity compared to molecular oxygen. Excessive accumulation of ROS disrupts intracellular homeostasis, potentially leading to the inactivation of proteins or enzymes, and can inflict oxidative damage on DNA and lipids, a phenomenon termed oxidative stress [4–8]. In eukaryotic cells, ROS predominantly originate from the mitochondrial respiratory chain, with mitochondrial electron transport enzymes, the NADPH oxidase complex, and external stressors such as radiation and chemical exposure, acting as principal contributors to ROS generation [9, 10].

In the context of high protein-producing strains of *A. niger*, an additional concern arises when the endoplasmic reticulum (ER) is burdened beyond its capacity. This overload hinders proteins from achieving their native conformation, resulting in the accumulation of unfolded proteins within the ER lumen, a condition known as ER stress [11]. ER stress triggers the unfolded protein response (UPR), a critical adaptive mechanism. Within the ER, the major enzymatic players in ROS production are protein disulfide isomerase (*PDI*) and ER oxidoreductin (*ERO*), which are instrumental in the formation of protein disulfide bonds [12]. *ERO* facilitates the transfer of electrons from *PDI* to the ER lumen through flavin adenine dinucleotide (FAD)-dependent reactions. Through a series of thiol-disulfide exchange reactions, electrons are transferred from *PDI* to molecular oxygen, leading to the incomplete reduction of oxygen and the consequent formation of superoxide anion radicals. These radicals may then be converted to hydrogen peroxide (H_2_O_2_) or other ROS, culminating in oxidative stress induced by protein folding within the ER [4, 13]. Notably, there is a significant correlation between the UPR and oxidative stress triggered by ROS, highlighting the intricate relationship between protein folding, oxidative stress, and cellular homeostasis in *A. niger* [14].

In fungi, diverse strategies have been elucidated to mitigate the detrimental effects of ROS, with *Saccharomyces cerevisiae* serving as the model organism where these mechanisms are most comprehensively characterized. *S. cerevisiae* employs a bifurcated antioxidant defense system, encompassing both enzymatic and non-enzymatic strategies [15]. The non-enzymatic defense apparatus primarily comprises antioxidant molecules that neutralize ROS or stabilize free radicals, thereby averting oxidative harm to biomolecules. Key antioxidant constituents include GSH [16, 17], ascorbic acid (vitamin C), and tocopherol (vitamin E), among others. These molecules engage with ROS or free radicals to prevent their interaction with biomolecules, safeguarding cellular integrity from oxidative damage. Conversely, the enzymatic defense system is predicated on the electron transfer among antioxidant enzymes to eradicate ROS. Predominant antioxidant enzymes include catalase (CAT), glutathione peroxidase (GPX), and superoxide dismutase (SOD). In the context of elevated intracellular ROS levels inducing oxidative stress, the antioxidative stress transcription factors *Yap1* and *Skn7* orchestrate a coordinated response [18, 19]. *Yap1*, upon activation, translocates to the nucleus to upregulate the expression of a cadre of antioxidant enzymes, such as SOD and GPX, in addition to genes integral to the glutamatergic exchange system and the thioredoxin reduction system, including thioredoxin reductase (*Trr1)* and glutathione reductase (*Glr1*). The genes products thus generated contribute to maintaining the cellular redox state [20, 21]. *Skn7* primarily addresses hydrogen peroxide-induced stress, activating genes implicated in hydrogen peroxide detoxification (e.g., catalase [CAT]) and thioredoxin reductases [22, 23]. Additionally, *Skn7* plays roles in cell wall synthesis and repair, as well as cell cycle regulation [24].

In this study, we used the antioxidant system in *S. cerevisiae* and protein structure comparison to understand the oxidative stress response mechanisms in *A. niger*. To better investigate these systems, we created multiple functional modules, including a NADPH regeneration module, a glutaredoxin system, a GSH synthesis module, and a transcription factor regulatory module. Our comprehensive analytical technique aided in the discovery of key genes involved in GSH metabolism, which are important in conferring oxidative stress resistance and increasing protein production in *A. niger.* This integrative technique not only offers light on eukaryotic oxidative stress regulation, but also identifies prospective targets for increasing protein yield in industrial microbes.

## Material and methods

### Strains, reagents, and culture conditions

Laboratory-conserved *A. niger* SH-1 (Δ*pyrG*) (The strain of *A. niger* SH-1 was derived from the strain of *A. niger* CICC2462 by classical mutagenesis.) serves as a host for gene expression. All strains were grown in Czapek-Dox media (CD) (including 0.05% agar powder). Before transformation, *A. niger* was cultured in the Dextrose Peptone Yeast extract media (DPY), and shake flask fermentations were conducted utilizing a specific fermentation medium [25]. For detailed compositions of all media used, please refer to the Supplementary Material (Table S1). Sterilization of all materials was achieved through autoclaving at 115 °C for 20 min. The *A. niger* employed in our study is cataloged in Table 1. Cultivation conditions for all strains involved growth at 30 °C in CD medium for initial culture, followed by incubation in either DPY medium or the designated fermentation medium under a constant agitation of 220 rpm.

**Table 1:** *A. niger* used in this study

Strains	Parental strain	Description	Another name
P-WT	SH-1	ΔpyrG	P-WT
WT	P-WT	pyrG	WT
OEAn03	P-WT	An03g05090	N1(An03)
OEGlr1	P-WT	An03g03660	GS1(Glr1)
OEGrx1	P-WT	An04g00150	GS2(Grx1)
OEGrx2	P-WT	An15g03220	GS3(Grx2)
OEGrx3	P-WT	An08g00600	GS4(Grx3)
OEGrx4	P-WT	An18g04790	GS5(Grx4)
OEGSH1	P-WT	An02g09720	G1(GSH1)
OEGSH2	P-WT	An09g03030	G2(GSH2)
OEMetG	P-WT	An14g00930	G3(MetG)
OEMecB	P-WT	An16g08720	G4(MecB)
OEYap1	P-WT	An11g07980	T1(Yap1)
OEPrx	P-WT	An04g00120	T2(Prx)
OEGpxA	P-WT	An02g08110	T3(GpxA)
OESodC	P-WT	An07g03770	T4(SodC)
OESod2	P-WT	An04g04870	T5(Sod2)
OESodB	P-WT	An01g12530	T6(SodB)
OEHacA	P-WT	An01g00160	U1(HacA)
OEPdiA	P-WT	An02g14800	U2(PdiA)
OEEroA	P-WT	An16g07620	U3(EroA)
OEGr&An	OEGlr1	oeGlr1,oeAn03	C1(Gr&An)
OEGr&Ya	OEGlr1	oeGlr1,oeYap1	C2(Gr&Ya)
OEGr&Ya&An	OEGlr1	oeGlr1,oeYap1,oeAn03	C3(Gr&Ya&An)

*oe* overexpression, *∆ *knockout

### Construction of recombinant plasmids and construction of recombinant strains

The plasmids for expression cassettes and CRISPR/Cas9 utilized in this study are delineated in the Supplementary Material (Fig. S1). The repair template's open reading frame was synthesized using the genomic DNA of the P-WT strain as the template for the expression cassette vector. This template was amplified employing PrimeSTAR® Max DNA Polymerase (Takara, Japan). Subsequent ligation of the vector was performed using the Seamless Assembly cloning kit (CloneSmarter, USA), adhering strictly to the guidelines provided by the manufacturer. The resultant vector was then introduced into *E. coli Mach1 T1* cells, and plasmids were isolated utilizing the Plasmid Extraction Kit (Magen, Guangzhou, China) before the transformation process. For detailed visualization of the cas9 plasmid pFC332-02 g construction, targeting the integration site, refer to the schematic diagram presented in the Supplementary Material (Fig.S1). The integration of the *A. niger* genome, devoid of any labels, was executed as follows: the initial strain was converted into protoplasts through enzymatic digestion. These protoplasts were then meticulously washed, and a combination of pFC332-02 g and the repair template was introduced at a molar ratio of 1:10 (Fig. S2). Screening of the transformants was conducted on plates infused with thaumatin. The integration of tags into the genome was facilitated by the addition of ribonucleoprotein complexes (RNPs) [26] along with 50–100 μg of a repair template (Fig. S2). Given that the repair template is pre-tagged with *pyrG* (The source of this tagged gene is *Aspergillus nidulans* FGSC A4 and the Fungi DB accession number is AN6157.), transformants were efficiently screened utilizing hypertonic CD plates [27]. In this study, the genomic locus targeted by the RNPs is delineated between genes An17g00730 and An17g00740. Concurrently, the insertion site for the plasmid pFC332-02 g is demarcated between genes An02g08020 and An02g08030. The guide RNAs (gRNAs) (Table S2) specific to these sites were meticulously predicted and designed employing the CRISPR RGEN Tool (available at http://www.rgenome.net/cas-offinder/), with the last access dated December 15, 2022.

### Assessment of phenotypic responses under exogenous pressure

The strains were incubated in a CD liquid medium devoid of agar at a constant temperature of 30 °C for a duration ranging from 7 to 14 days. Subsequently, a specified volume of the culture with an optical density (OD_600_) of 1 was homogenized. From this homogenate, 10 μl aliquots were inoculated onto CD agar plates that had been pre-supplemented with varying concentrations of the exogenous agent. The resultant phenotypic manifestations and growth patterns were meticulously observed following static incubation for three days at 30 °C.

### Quantitative analysis of intracellular ROS and GSH levels

Mycelia of *A. niger* in their exponential growth phase were harvested through filtration and subjected to multiple washes with both water and phosphate-buffered saline (PBS) solutions, respectively, to ensure complete removal of the growth medium. Mycelia were dried overnight at 60 ℃ [19]. To facilitate the detection of intracellular ROS, a precise mass of 0.0500 g of mycelium was measured. The DCFH-DA probe, obtained from Solarbio's ROS detection kit (Solarbio, Beijing, China), was diluted 2000-fold for the assay. Fluorescence microscopy was employed for detection, with excitation and emission wavelengths set at 488 nm and 525 nm, respectively. For the quantification of GSH, the DTNB (5,5'-dithio-bis-(2-nitrobenzoic acid)) method was employed. Mycelia were dried overnight at 60 ℃. Specifically, 0.1000 g of the mycelium was accurately weighed and homogenized in 1 ml of GSH Content Assay Reagent 1 (Solarbio, Beijing, China) using liquid nitrogen. Following thorough grinding, the sample was centrifuged at 8000 *g* for 10 min at 4 °C. Subsequently, Reagent 2 and Reagent 3 from the kit were added in sequence, and the mixture was allowed to stand at room temperature for 2 min before the absorbance was measured at 412 nm. The experiments were performed in triplicate.

### Determination of glucoamylase activity and total protein level

The strains were cultured in a fermentation medium at 30 °C with an agitation speed of 220 rpm for 7 days. Following incubation, the cultures were centrifuged at 12,000 rpm for 10 min at ambient temperature to collect the clear supernatant containing the fermentation broth. The activity of glucoamylase in the supernatant was quantitatively assessed using a spectrophotometric method, employing p-nitrophenyl-α-D-glucopyranoside (pNPG) as the substrate [28]. Total extracellular proteins in culture supernatants were determined using the BCA Protein Assay Kit (GBCBIO, Guangzhou, China) according to the manufacturer’s manual. Enzyme activity assays were performed in triplicate.

### RNA extraction and transcriptome sequencing

The mycelium of *A. niger* was harvested at the designated index period, employing the methodology delineated earlier, and was immediately frozen in liquid nitrogen. After thorough pulverization, total RNA was isolated using the HiPure Fungal RNA Kit (Magen, Guangzhou, China), in strict accordance with the manufacturer's protocols. The isolated RNA was then reverse-transcribed utilizing the PrimeScript RT-PCR Kit (TaKaRa, Japan) [29]. Transcriptome sequencing was conducted on the Illumina HiSeq™ platform at Sangon Bioengineering (Sangon, Shanghai, China). The generated sequencing datasets underwent rigorous quality evaluation and analysis to ensure the integrity and reliability of the transcriptomic insights.

## Results

### Engineering the antioxidant defense metabolism to detoxify ROS in *A. niger*

**Fig. 1:**
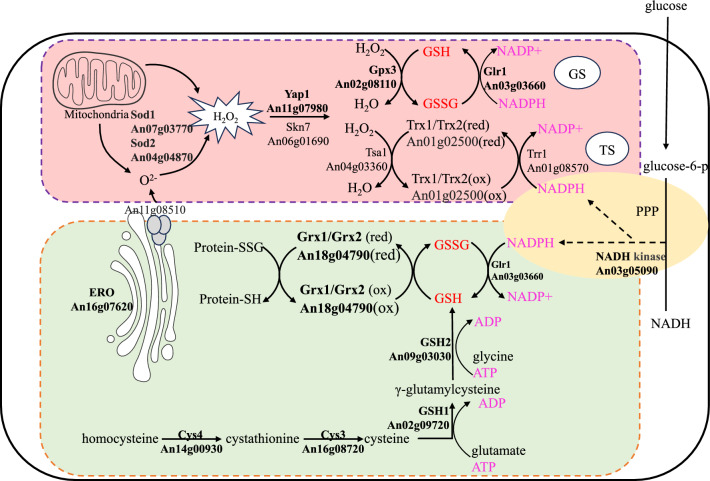
Antioxidant defense metabolism to detoxify ROS in response to intracellular oxidative stress in *A. niger*. The function genes of the antioxidant defense metabolism in the genome of *A. niger* CBS513.88 were annotated from homologous genes of *S. cerevisiae*'s antioxidant system by the method of the protein BLAST. Simplified illustration of the antioxidant defense mechanism of *A. niger* in response to intracellular oxidative stress which is generated from the source of ROS in the endoplasmic reticulum and mitochondria. The antioxidant defense metabolism divided into four modules: NADPH regeneration, glutaredoxin system, GSH synthesis system, and transcription factor regulation. The NADPH regeneration module principally supports the provision of NADPH in *A. niger* through the pentose phosphate route and NAD kinase. An11g07980 (*Yap1*) and An06g01690 (*Skn7*) activate the system (GS) and thioredoxin system (TS), which coordinate transcriptional regulation. The genes overexpressed in this present study are highlighted in bold

To study antioxidant defense metabolism to detoxify ROS in response to intracellular oxidative stress in *A. niger*, we annotated the function genes of the antioxidant defense metabolism of *A. niger* from homologous genes of *S. cerevisiae*'s antioxidant system by the method of the protein BLAST (Fig. 1). We categorized the antioxidant system of *A. niger* into several distinct components, which can be simplified as follows: ROS generated in the ER or mitochondria are first converted into hydrogen peroxide by SOD. This hydrogen peroxide then activates the transcription factor *Yap1* (An11g07980). *Yap1*, in turn, induces the expression of its downstream target proteins, including peroxidase and SOD. Additionally, *Yap1* regulates both the GSH and thioredoxin systems. Within the glutathione system, GSH plays a pivotal role in maintaining ER homeostasis. Therefore, we included the GSH synthesis pathway in our analysis, which encompasses *GSH1* (An02g09720), *GSH2* (An09g03030), *MetG* (An14g00930), and *MecB* (An16g08720). The systems regulated by *Yap1* are dependent on NADPH for their activity, represented in the figure by NAD kinase (An03g05090). Consequently, we segmented this system into the following components: NADPH regeneration engineering module, glutaredoxin system, GSH synthesis engineering module, and transcription factor module.

### Engineering the antioxidant defense metabolism to improve protein secretion in *A. niger*

**Fig. 2:**
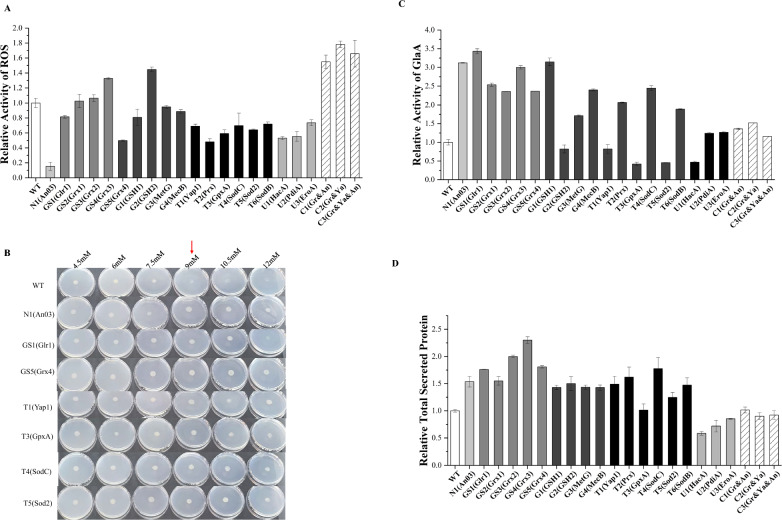

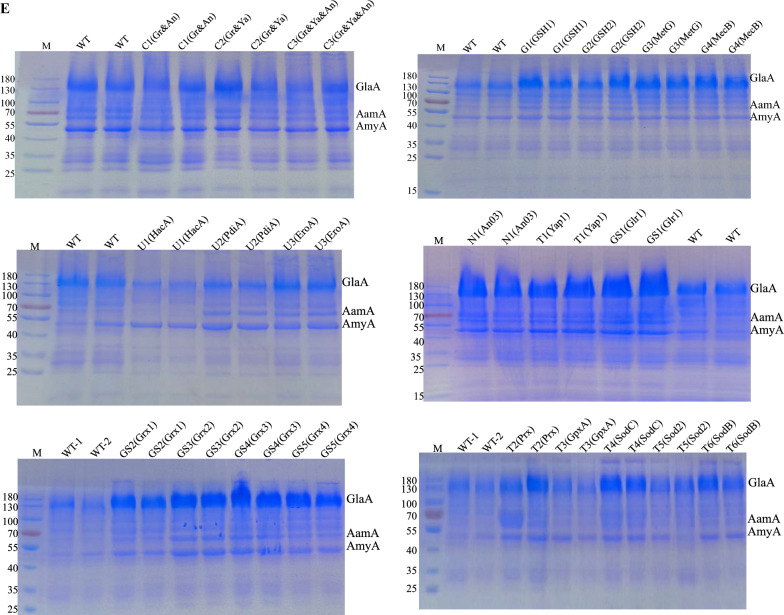
Engineering antioxidant defense metabolism in *A. niger* increased the amount of protein secreted. **A** The intracellular ROS levels in the *A. niger* and its mutants were determined with Solarbio's ROS detection kit and the DCFH-DA probe. The ROS level in the WT was used as the control and the ROS levels of the mutants were compared with that of the WT. The FungiDB accession numbers for these strains are listed in Table 1. **B** The growth morphology of mutant strains on agar plates at series H_2_O_2_ concentrations ranging from 4.5 to 12 mM, and the WT as control, demonstrating the differential oxidative stress resilience conferred by modified modules. A red arrow has been added at the 9 mM concentration in the figure to highlight the significant growth differences observed between the wild type (WT) and the overexpression strains at this concentration. **C** The glucoamylase (GlaA) is the main representative of extracellular secreted proteins in *A. niger*. The glucoamylase enzyme activity for all strains was measured using the p-nitrophenyl-β-D-glucopyranoside (pNPG) assay. The activity of the WT was used as a control for normalization. **D** Total secreted protein levels were determined for all mutant strains using the BCA method and were set at 1 for the WT. **E** The amount of the secreted proteins from *A. niger* mutants was determined using sodium dodecyl sulfate–polyacrylamide gel electrophoresis (SDS-PAGE). In the SDS-PAGE figure, 10 µl of sample was loaded onto the gel when the number of samples was less than 10. Conversely, when the number of samples exceeded 10, 5 µl of the sample was loaded onto the gel.The SDS-PAGE plot of Marker using PageRuler 26616 (Thermo Fisher) with molecular weights ranging from 180 to 15 kDa. The gels show the locations of important secreted proteins from *A. niger*, including glucoamylase (GlaA), acid amylase (AamA), and neutral amylase (AmyA). The numerical identifiers before the gene names for overexpressing strains identify the respective modules: 'N' for the NADPH regeneration engineering module, 'GS' for the glutaredoxin system, 'G' for the glutathione synthesis engineering module, 'T' for the transcription factor regulation module, and 'U' for the UPR module

To enhance the ability of *A. niger* to detoxify ROS, we focused on strengthening key metabolic modules, including the glutaredoxin system, NADPH regeneration, transcription factor regulation, and UPR. This resulted in the generation of mutants N1 to U3, each overexpressing specific functional genes as detailed in Table 1. By quantifying the endogenous ROS levels in the strains, we observed a significant reduction in ROS content in the mutants. Particularly, the N1 mutant overexpressing NAD kinase showed ROS levels that were 15% of those of the wild type (WT) (Fig. 2A). Mutants related to glutathione biosynthesis (G1–G4), with overexpression of gamma-glutamylcysteine synthetase *GSH1* in the G1 mutant, showed an 18% reduction in ROS levels compared to the WT. Mutants related to the glutaredoxin system (GS1–GS5), with overexpression of glutathione reductase *Glr1* (An03g03660) in the GS1 mutant, showed a reduction of approximately 52% in ROS levels compared to the WT. Additionally, the T1 mutant overexpressing the transcription factor *Yap1* showed a 31% reduction in ROS levels compared to the WT. It is worth noting that the overexpression of glutaredoxin had a minor impact on ROS regulation, with OEGrx2 (mutant strains overexpressing An15g03220) leading to an increase in ROS levels. Mutants related to the UPR mechanism showed a 26–47% reduction in ROS levels compared to the WT (Fig. 2A). Furthermore, experiments involving mutants with co-overexpression of glutathione reductase *Glr1*, NAD kinase (An03g05090), and transcription factor *Yap1* were conducted. Evaluation of ROS levels in these recombinant strains showed that compared to the WT, these strains had ROS levels that were 1.55 to 1.78 times higher (Fig. 2A).

To assess the strains' ability to detoxify ROS, the growth morphology on solid media supplemented with various exogenous oxidative stressors was measured (Fig. 2B). Mutants were cultured on solid CD media containing varying concentrations of H_2_O_2_ and methyl menadione sodium bisulfite (MSB). While the WT and overexpressing strains exhibited different growth responses to these stressors, overall, mutants showed enhanced resistance, performing better than the WT under certain conditions. Specifically, the WT exhibits normal growth at a 6 mM H_2_O_2_ concentration, but its growth is compromised at higher concentrations. Notably, at the 9 mM concentration, significant growth differences are observed between the WT and the overexpression strains. Similarly, the WT experienced growth inhibition at concentrations ranging from 1.5 to 12 mM MSB, while overexpressing strains showed improved growth performance, albeit with significant inhibition compared to the WT (Fig. S3). We assayed intracellular ROS levels and cultured the strain on solid plates with externally added oxidative stress to identify genes suitable for modulating the intracellular redox state of *A. niger*, specifically focusing on the transcription factor *Yap1* and the NAD kinase *An03*.

To investigate the impact of the enhanced antioxidant capacity of *A. niger* on its protein secretion, we evaluated glucoamylase activity across all strains (Fig. 2C). The total secreted protein content of all mutant strains was examined using the BCA method (Fig. 2D), and the secreted protein levels were characterized by SDS-PAGE (Fig. 2E). Various overexpression strains demonstrated distinct abilities in modulating protein secretion dynamics. Compared to the WT, overexpression of NAD kinase increased glucoamylase activity to 312% of the WT and elevated total secretory protein levels to 175% of the WT. Notably, the overexpression of *Grx1* (An04g00150) and *Grx4* (An18g04790) led to a reduction of 18% in glucoamylase levels, while simultaneously elevating total protein secretion levels by 31% and 39%, respectively. Particularly noteworthy was the profound impact of *Glr1* (An03g03660), which not only elevated glucoamylase activity to 3.43-fold of the WT, but also augmented total protein levels to 1.88-fold of the WT, demonstrating the most significant effect among all genes studied. Elevated expression of the glutathione synthesis module resulted in increased glucoamylase enzyme activity (Fig. 2C), with MetG (An14g00930) increased glucoamylase enzyme activity by 200% and total protein levels by 69% compared to the WT. Transcription factor *Yap1* and its regulatory proteins demonstrated differential effects on glucoamylase enzyme activity and total protein levels upon overexpression. *Yap1* overexpression resulted in a 3.15-fold increase in glucoamylase activity of the WT and a 1.68-fold increase in total protein of the WT. Conversely, *GpxA* (An02g08110) and *Sod2* (An04g04870) significantly reduced glucoamylase activity while elevating total protein to 1.25-fold and 1.27-fold of the WT, respectively. Mutant strains with up-regulated UPR pathway-related genes exhibited no significant alterations in glucoamylase activity or total protein secretion levels compared to the WT (Fig. 2C, D). In contrast, OEHacA (mutant strains overexpressing An01g00160) displayed decreased secreted protein levels, with 53% of glucoamylase activity and 7% of total protein compared to the WT. Our findings revealed that the co-expression of a substantial number of significantly up-regulated genes resulted in a multimutant strain with a slight increase in glucoamylase enzyme activity. When *Glr1* and *Yap1* were co-expressed, glucoamylase enzyme activity increased to 152% of the WT, and total secretory protein levels increased to 127% of the WT (Fig. 2C, D). Despite this, this combination was one of the most effective among those tested. In contrast, when all three genes were simultaneously overexpressed, glucoamylase levels increased to 116% of the WT, and total secretory protein levels increased to 118% of the WT. Our findings illustrate that reducing intracellular ROS levels can significantly enhance the secretion levels of total protein in *A. niger* through the overexpression of individual genes. Notably, the overexpression of *Glr1* emerged as the most efficacious, amplifying glucoamylase enzyme activity by 243% and total protein secretion by 88%. The overexpression of *An03* and *Yap1* also yielded substantial enhancements. Nonetheless, the simultaneous co-expression of these three genes did not yield the anticipated reduction in ROS levels. Instead, it led to a substantial decline in protein secretion levels, markedly inferior to the outcomes observed with the overexpression of individual genes.

### Studies on GSH changes during protein synthesis and secretion in *A. niger*

**Fig. 3:**
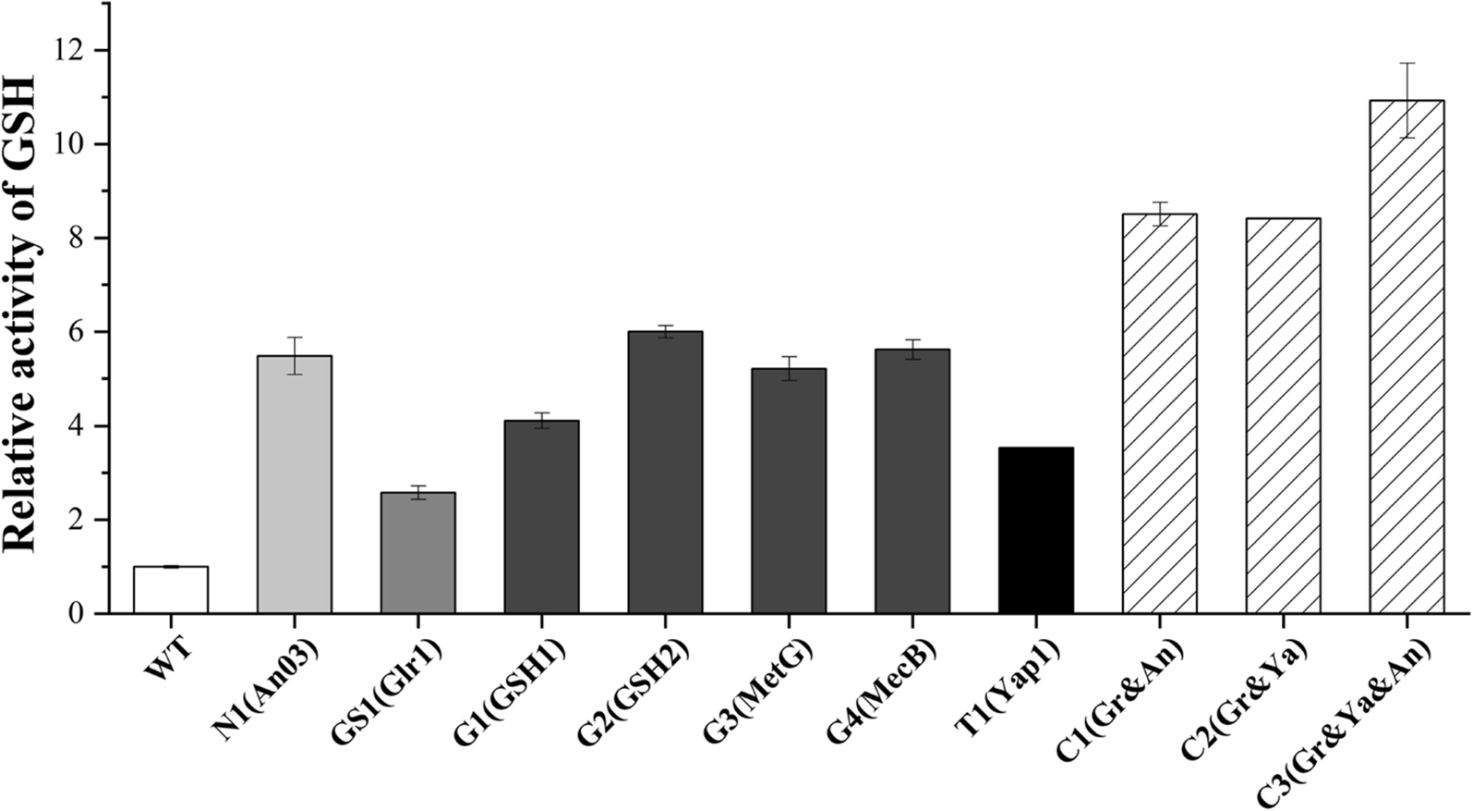
Comparative analysis of intracellular GSH content in recombinant *A. niger* strains. This graph depicts the intracellular GSH concentration of strains overexpressing *Glr1* (OEGlr1), *Yap1* (OEYap1), *An03* (OEAn03), and their respective multi-gene combinations, as well as strains designed to overexpress *GSH1* (OEGSH1), *GSH2* (OEGSH2), *MetG* (OEMetG), and *MecB* (OEMecB). The GSH content of the WT is normalized to 1, which serves as a baseline for comparison

To elucidate the role of GSH content in the protein secretion capability of *A. niger*, we investigated the intracellular GSH content in both high-expression strains (N1, GS1, and T1) and mutant strains overexpressing GSH synthase (G1-G4). Our analysis revealed that a moderate elevation in GSH content facilitates protein secretion, whereas an excessive augmentation adversely affects the secretion levels. We found that overexpression of *An03* increased the intracellular GSH content of *A. niger* up to 5.49-fold of the WT, overexpression of *Glr1* increased GSH content up to 2.58-fold of the WT, and overexpression of *Yap1* resulted in a GSH content up to 3.54-fold of the WT (Fig. 3). However, intracellular GSH content was significantly increased in the multiple combination strains of these three genes, with GSH content exceeding 8.41-fold of the WT in all three multiple combination strains, and reaching 10.93-fold of the WT in the strain expressing all three genes simultaneously. Surprisingly, the significant increase in GSH did not promote protein secretion (Fig. 2C, D), nor did it reduce ROS levels (Fig. 2A), but instead elevated ROS levels. Overexpression of *GSH1* in the GSH synthesis pathway increased intracellular GSH content up to 4.11-fold of the WT, while overexpression of the remaining three genes, *GSH2*, *MetG*, and *MecB*, resulted in GSH increases up to 5.93-fold, 5.21-fold, and 5.62-fold, respectively, of the WT. Our findings indicate that in *A. niger*, both glucoamylase activity and overall protein secretion levels in the SH-1 strain were significantly enhanced upon elevating the GSH content to 2.58–5.93 times that of the WT. However, our data show that when GSH levels were elevated to more than 8.42 times that of the WT, both glucoamylase enzyme activity and total secretory protein levels were significantly reduced, despite a slight increase compared to the WT. This observation suggests that excessive GSH concentrations may have a deleterious effect on enzyme activity and protein secretion, indicating an optimal threshold for GSH that supports cellular function without hindering biochemical processes.

### Genome-wide transcriptional differences under reduced intracellular oxidative stress

**Fig. 4:**
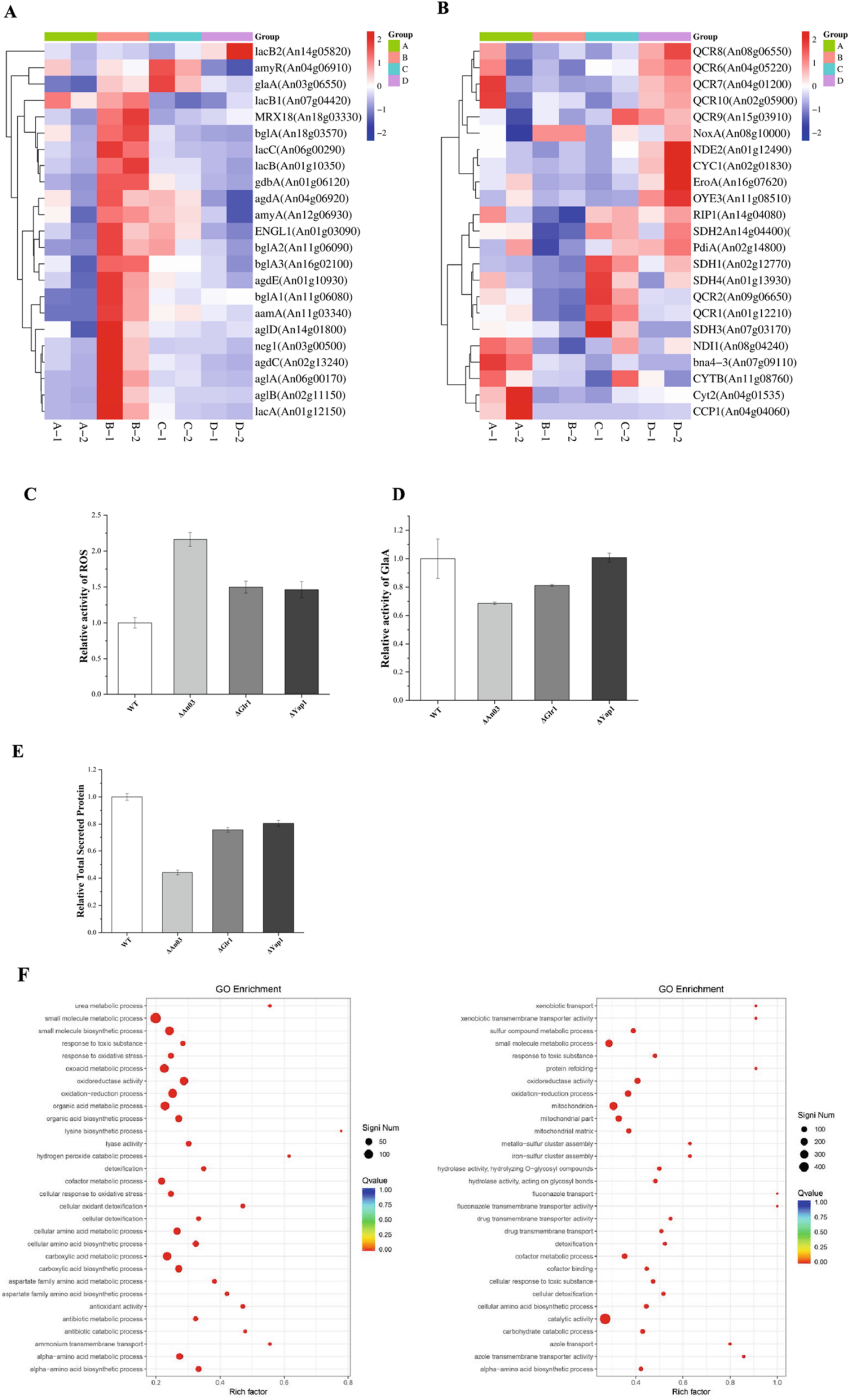
RNA-Seq analysis of WT, OEGlr1, OEAn03 and OEYap1. **A** Delineates the expression profiles of common secreted proteins in *A. niger*, such as *glaA*, *amyA*, and *aamA*. **B** Delineates the expression profiles of ROS-producing genes in *A. niger*. **C** Intracellular ROS levels in *A. niger* strains with the knockout of *Glr1*, *An03* and *Yap1*. **D** Glucoamylase activity of *A. niger* strains with the knockout of *Glr1*, *An03* and *Yap1*. **E** Total secretory protein levels in *A. niger* strains with the knockout of *Glr1*, *An03* and *Yap1*. **F** Shows the enrichment in OEYap1 and OEGlr1, with the vertical axis denoting function annotation information and the horizontal axis representing the Rich factor. The Rich factor is determined by dividing the number of differentially expressed genes ascribed to a certain function by the total number of genes annotated to that function. The color intensity of each dot is proportional to the Q-value magnitude; a lower Q-value produces a color closer to red. Furthermore, the dot size represents the number of differentially expressed genes for each function. To ensure clarity, only the top 30 Gene Ontology (GO) terms with the highest enrichment degree were plotted

To elucidate the transcriptional alterations associated with reduced intracellular oxidative stress, we conducted transcriptome sequencing of the WT and genetically modified strains of *A. niger*: overexpressors of *Glr1* (OEGlr1), *An03* (OEAn03), and *Yap1* (OEYap1). Previous studies have demonstrated that alleviating intracellular oxidative stress enhances both glucoamylase activity and the total secretion of proteins in *A. niger* SH-1. Accordingly, we analyzed the transcriptional profiles of common secretory proteins (enzymes) in *A. niger* based on their Fragments Per Kilobase per Million (FPKM) values (Fig. 4A). Enzymes analyzed included *amyR* (An04g06910), *glaA* (An03g06550), *amyA* (An12g06930), *aamA* (An11g03340), and *agda* (An04g06920). Our findings show that overexpression of *Glr1* markedly increases the transcription levels of these proteins. Similarly, OEAn03 significantly upregulates the transcription of *glaA*, a pattern also observed with OEYap1. Furthermore, we identified genes in *A. niger* associated with ROS production (Fig. 4B), including components of mitochondrial electron transport chain complexes I–III (including An08g04240, An02g12770, An02g01830, etc.), and their precursors or cofactors, NADPH oxidase (An11g08510, An08g10000), endoplasmic reticulum oxidoreductase (An16g07620), and disulfide bond isomerase (An02g14800) (Fig. 4B). Transcriptional profiling indicated that *Glr1* overexpression leads to a substantial downregulation of ROS-generating genes. Conversely, overexpression of NAD kinase diminishes transcript levels of genes associated with complexes I and III, while paradoxically increasing those of complex II. Overexpression of *Yap1* upregulates transcript levels of complexes I-III without inhibiting ROS production at the transcriptional level. From these transcriptional profiles, it is evident that reducing intracellular oxidative stress enhances protein transcription. To further investigate, we generated knockout strains for *Glr1*, *An03*, and *Yap1*, designated as ΔGlr1, ΔAn03, and ΔYap1, respectively. Post-fermentation determination ((Fig. 4C–E)) revealed that ΔAn03 exhibited the most pronounced changes, with a 31% reduction in glucoamylase activity and a 56% decline in total secreted protein levels. Additionally, the ROS level in ΔAn03 increased by 116%. The outcomes in the other two knockout strains were more similar; however, the glucoamylase activity of ΔYap1 remained comparable to that of the WT.

The Gene Ontology (GO) analysis revealed significant functional enrichments for OEYap1 and OEGlr1, as depicted in the scatter plots (Fig. 4F). Specifically, in the comparison between OEYap1 and the WT, a notable enrichment was observed in pathways such as GO:0044281 (small molecule metabolic process), along with oxidoreductase activity, oxidation–reduction process, carboxylic acid metabolic process, oxoacid metabolic process, and organic acid metabolic process, indicating a high number of differentially enriched gene sets in these pathways. Conversely, OEGlr1 showed a distinct enrichment profile, with a higher prevalence in catalytic activity, mitochondrial localization, and small molecule metabolic processes. This enrichment highlights the potential influence of these genetic alterations on *A. niger*'s metabolic landscape, particularly in terms of increasing its capacity for small molecule metabolism and oxidation–reduction activities. Catalytic activity and small molecule metabolic pathways were all significantly enriched in the OEGlr1. Comparable findings were observed with OEAn03, with comprehensive results available in the Supplementary Material (Fig. S4). Collectively, our findings suggest that overexpression of *Yap1* mitigates ROS through the activation of antioxidant downstream proteins, differing fundamentally from the mechanism employed by *Glr1*. Specifically, *Glr1* overexpression decreases ROS levels by downregulating genes involved in ROS production. Moreover, *Glr1* significantly reduces the mitochondrial energy supply, which is compensated by an enhancement of the cell's overall catalytic activity and the metabolism of small molecules. In contrast, overexpression of *An03* appears to be linked with the stimulation of intracellular vesicular transport and secretion mechanisms.

## Discussion

*A. niger* is renowned for its exceptional capacity to produce industrial enzymes, serving as an exemplary fungal cell factory. The augmentation of protein production, with a particular emphasis on glucoamylase, has traditionally focused on strategies such as gene amplification, the deployment of strong promoters, and the engagement of molecular chaperones [30–32]. In employing the aforementioned strategy, there is an inherent increase in stress on the ER, as protein folding constitutes a critical juncture in the secretory pathway. Only proteins that are correctly folded can be secreted extracellularly, whereas misfolded proteins are targeted for degradation into amino acids via the ER-associated degradation (ERAD) pathway [33, 34]. Protein folding demands substantial quantities of redox agents, the utilization of which generates ROS as a byproduct. Excessive accumulation of ROS can induce ER stress and trigger the UPR, which strives to restore ER functionality by diminishing protein synthesis, enhancing protein degradation, and facilitating protein folding. However, the activation of the UPR might also contribute to further ROS production [35, 36], and sustained ER stress can lead to cell death if the UPR is unable to rectify the protein folding disruptions, a situation potentially exacerbated by ROS accumulation. Previous studies, such as those by Marizela et al., have successfully mitigated the metabolic burden and cellular stress associated with high oxidative loads during protein folding [37]. This was achieved by modulating the antioxidant capacity in *Pichia pastoris*, thereby reestablishing suitable redox conditions and enhancing secretory capacity. Building on these insights, the present study utilized the highly glucoamylase-secreting *A. niger* SH-1 strain (Δ*pyrG*) as the foundational model. This choice was driven by our aim to investigate whether reducing intracellular oxidative stress could amplify the strain’s protein secretion capabilities. In this study, we identified and described a cohort of genes implicated in the oxidative stress response of *A. niger* using comparative analysis of protein structures. Current studies indicate that under oxidative stress conditions, such as the presence of H₂O₂, the transcription factor *Yap1* undergoes conformational changes to form disulfide bonds and aggregates in the nucleus. This aggregation facilitates the activation of genes encoding downstream antioxidant enzymes and molecular chaperones, including superoxide dismutase and glutathione peroxidase. In addition to the aforementioned downstream proteins, *Yap1* also orchestrates the regulation of the glutaredoxin and thioredoxin systems. The glutaredoxin system utilizes NADPH to convert GSSG to GSH. GSH can function within the mitochondria and endoplasmic reticulum. Furthermore, GSH plays a dual role by directly interacting with ROS in a non-enzymatic manner, highlighting the importance of the GSH synthesis pathway in cellular defense mechanisms. So, we deconstructed it into four distinct modules: NADPH regeneration engineering, glutaredoxin system, GSH synthesis engineering, and transcription factor regulation. Additionally, given the significant correlation between the UPR and the antioxidant system, we also investigated the UPR module. We systematically examined the impact of overexpressing these genes in recombinant strains, examining multiple parameters including intracellular ROS levels, growth under oxidative stress, protein production, and GSH level.

Initial tests focused on intracellular ROS, which revealed that overexpression often reduced ROS levels. Remarkably, most of the recombinant strains succeeded in diminishing intracellular ROS levels, with the strain overexpressing NAD kinase (*An03*) achieving the most significant reduction (Fig. 2A). NAD kinase is essential for maintaining intracellular levels of reduced NADPH by catalyzing the conversion of NAD^+^ to NADPH, one of the major intracellular reductants that plays a central role in the intracellular antioxidant defense system.

Interestingly, ROS levels increased in the OEGrx2, despite its close homolog possessing glutathione disulfide oxidoreductase activity, yet the precise mechanism remains elusive. Additionally, we identified that genes associated with the UPR contribute to the reduction of ROS levels, potentially due to a decreased need for protein folding, thereby conserving ATP for disulfide bond formation, because the accurate folding of nascent polypeptides is dependent on the ATP-fueled actions of molecular chaperones and disulfide bond isomerases [38]. This insight suggests a link between reduced oxidative stress and the optimization of protein folding processes within the cell. *A. niger* B1-D was exposed to sub-lethal concentrations of hydrogen peroxide, which resulted in growth retardation as well as upregulation of antioxidant defenses such as superoxide dismutase, catalase, glutathione peroxidase, and glutathione reductase [39]. Cultivation of the recombinant strains on solid media containing hydrogen peroxide and MSB revealed a significant increase in resilience to exogenous oxidative stress compared to the WT counterparts, highlighting the protective benefit supplied by the overexpressed antioxidant genes. Notably, the OEYap1 distinguished itself by demonstrating the most pronounced stress resistance, as demonstrated by its performance on selective media under stress conditions.

We explored the protein secretion capabilities of various overexpressed strains, given the extensive and complex nature of secreted proteins by *A. niger*. We selected the SH-1 strain, known for its high glucoamylase production, as a representative marker for assessing secreted enzyme activity. Our findings indicated a reduction in enzyme activity for the strain overexpressing the UPR transcription factor *HacA*, aligning with previous reports [40]. Overexpression of *PdiA* and *EROA* did not markedly enhance enzyme activity. Among the components of the glutathione reduction system [41], *Glr1* and *An03* emerged as significant contributors to enzyme activity enhancement. *Glr1*, by utilizing NADPH as an electron donor, facilitates the reduction of GSSG to two GSH molecules, thereby replenishing GSH for its antioxidant functions [42]. The expression of *Glr1* has been demonstrated to safeguard proteins against ROS-induced oxidative damage by activating multiple pathways, thus enhancing both proteostasis and redox homeostasis in yeast cells [43]. Our laboratory's prior work has established that overexpression of *An03* notably boosts glucoamylase activity [28]. Remarkably, overexpression of *An03* at the amyA site of the *A. niger* genome resulted in a glucoamylase enzyme activity that was 1.7-fold that of the starting strain, altering the insertion site resulted in the glucoamylase activity reaching 312% of the WT, a finding corroborated by both transcriptomic analyses and protein gel electrophoresis. The transcriptome analysis revealed that the gene encoding glucoamylase, *glaA*, was transcribed at exceptionally high levels (see Fig. 4A). Despite this, the enzyme activity did not surpass that observed with overexpression of *Glr1*. This discrepancy may be attributed to the dynamics of gene copy number and cellular capacity. Specifically, introducing an additional copy of the *glaA* gene effectively enhances its transcriptional activity, potentially increasing the production of glucoamylase in *A. niger* [30]. However, overloading the host cell with excessive copies of *glaA* may inadvertently impede the overall production of glucoamylase [44], suggesting a complex interplay between gene dosage and cellular processing capabilities. This finding underscores the need to balance gene expression and cellular capacity to optimize enzyme production.

The constitutive co-overexpression of *PpYAP1* in *P. pastoris* significantly elevates the level of secreted recombinant proteins, due to the overexpression of this transcription factor [37]. *Yap1* overexpression affects GSH accumulation at both its biosynthesis and substrate availability levels [45]. Our current experiments show that constitutive overexpression of *Yap1* not only significantly increased protein secretion, but also effectively reduced intracellular levels of ROS and increased intracellular GSH concentrations. These findings emphasize *Yap1* overexpression's potential as a strategic tool for optimizing recombinant protein production in yeast systems, as it increases both the output of secreted recombinant proteins and the cellular antioxidant capacity. Enhancements in glutathione biosynthesis within brewer's yeast have previously been reported to bolster the resilience of lignocellulosic materials pre-treated for bioconversion, particularly against inhibitors [46]. In our study, overexpression of *Yap1* in *A. niger* enhanced the strain's resistance to oxidative stress, increased GSH content, and also enhanced protein secretion levels. By overexpressing the genes *GSH1*, *GSH2*, *MetG*, and *MecB* in the SH-1, we achieved a notable increase in glucoamylase activity, surpassing 2.35-fold that of the WT in the recombinant variant. Furthermore, our investigation into the synergistic effects of gene overexpression, we discovered that ROS levels were considerably higher in strains with multigene recombinations than those with single-gene overexpression. This significant increase in GSH levels in multigene recombinant strains modify the proteins' redox status. Such changes may aggravate oxidative stress, negatively impacting protein folding and stability (Fig. 2A). Furthermore, the likelihood of S-glutathionylation of proteins in these settings may have an impact on protein functionality, implying a complicated interplay between high GSH levels and protein biochemistry that requires additional exploration [41, 47–49].

Our findings revealed that adjusting intracellular GSH levels could significantly increase the secretory output of *A. niger* proteins. This optimization entails glutathione reductase (*Glr1*) enzymatically reducing GSSG to GSH while *An03* simultaneously provides NADPH to match the metabolic demands of this redox system, both of which are critical for the effective conversion of GSSG. Furthermore, expanding the GSH biosynthesis route is another promising method for increasing protein secretion. However, it is important to note that an excess of GSH over the ideal threshold causes a perceptible decrease in *A. niger* protein secretion, demonstrating the need for a balanced approach to adjusting GSH levels to attain maximum secretory efficiency. This finding underscores the delicate balance required in the redox environment for optimal protein folding and secretion in *A. niger*, highlighting the complex interplay between gene overexpression and cellular biochemical pathways [37].

## Conclusion

In this study, we successfully delineated genes involved in the conversion and synthesis of GSH by specifically overexpressing antioxidant genes, revealing their significant influence on protein secretion—most notably, the secretion of glucoamylase in the SH-1 strain. While direct GSH synthesis did not considerably lower ROS, GSH conversion genes significantly reduced intracellular ROS concentrations. *Glr1* proved to be the most effective of them, primarily targeting GSSG and so effectively altering the intracellular GSH/GSSG balance. This modification significantly boosted GSH availability, consequently reinforcing *A. niger*'s antioxidant defenses and increasing enzyme productivity through better protein production. Notably, *Glr1* overexpression increased glucoamylase activity significantly, demonstrating the value of targeted genetic interventions in fine-tuning biochemical pathways for enzyme synthesis in the industrial setting. Our findings highlight the critical importance of selective antioxidant gene alteration in increasing enzyme yields and establish the framework for future research into the genetic optimization of microbial strains for increased production of commercially important enzymes. This discovery has great potential for developing biotechnological applications and optimizing industrial operations.

### Supplementary Information

Supplementary Material 1. Table S1 The medium and its components used in this study. Table S2 The sgRNA used in this study. Figure S1 The plasmids for expression cassettes and CRISPR/Cas9 utilized in this study. Figure S2 Schematic representation of gene editing using CRISPR/Cas9 plasmid and RNP complexes. Figure S3 presents the growth patterns of both control and mutant strains on agar plates subjected to incremental concentrations of MSB, ranging from 1.5 to 12 mM. Figure S4 Significantly enriched GO scatter plots for OEAn03 and WT.

## Data Availability

All data generated or analyzed during this study are included in this published article and its Additional file.

## Abbreviations

WT: Wild type
ER: Endoplasmic reticulum
ROS: Reactive oxygen species
UPR: Unfolded protein response
MSB: Menadione sodium bisulfite
FPKM: Fragments per kilobase per million
