# Supplementary material for: Enhancement of protein production in Aspergillus niger by engineering the antioxidant defense metabolism
Source: Biotechnol Biofuels Bioprod. 2024 Jun 29;17:91. doi: 10.1186/s13068-024-02542-0 (PMC11218396; doi:10.1186/s13068-024-02542-0)
Supplement: Supplementary file 1 — Supplementary Material 1. Table S1 The medium and its components used in this study. Table S2 The sgRNA used in this study. Figure S1 The plasmids for expression cassettes and CRISPR/Cas9 utilized in this study. Figure S2 Schematic representation of gene editing using CRISPR/Cas9 plasmid and RNP complexes. Figure S3 presents the growth patterns of both control and mutant strains on agar plates subjected to incremental concentrations of MSB, ranging from 1.5 to 12 mM. Figure S4 Significantly enriched GO scatter plots for OEAn03 and WT. [file 13068_2024_2542_MOESM1_ESM.docx]

**Table S1** The medium and its components used in this study

| media | Components and their contents |
| --- | --- |
| Luria-Bertani (LB) | peptone 1% (w/v), yeast extract 0.5% (w/v), and NaCl 1% (w/v) |
| Czapek-Dox medium (CD) | glucose 2% (w/v), NaNO_3_ 0.3% (w/v), KCl 0.2% (w/v), MgSO_4_·7H_2_O 0.05% (w/v), KH_2_PO_4_ 0.1% (w/v), FeSO_4_·7H_2_O 0.001% (w/v), and Agar 0.05% (w/v) |
| The hyperosmotic CD medium | sucrose 35% (w/v), NaNO3 0.3% (w/v), KCl 0.2% (w/v), MgSO4-7H2O 0.05% (w/v), KH2PO4 0.1% (w/v), and FeSO4- 7H2O 0.001% (w/v) |
| DPY medium | glucose 2% (w/v), peptone 1% (w/v), yeast extract 0.5% (w/v), KH2PO4 0.5% (w/v), and MgSO4-7H2O 0.05% (w/v) |
| Flask-level fermentation medium | maltose 7%(w/v), peptone 2.5% (w/v), yeast extract 1.25% (w/v), KH_2_PO_4_ 0.1% (w/v), MgSO_4_·7H_2_O 0.05% (w/v), FeSO_4_·7H_2_O 0.03% (w/v), ZnCl_2_ 0.003% (w/v), CaCl_2_ 0.002% (w/v), MnSO_4_·7H_2_O 0.0009% (w/v) , K_2_SO_4_ 0.2% (w/v) and pH was adjusted to 5.5 by 1 M HCl. |

**Table S2** The sgRNA used in this study

| Site | Sequences (5'→3') |
| --- | --- |
| Between genes An17g00730  And An17g00740 | ACGTAAGCAAAACAAATTCG |
| Between genes An02g08020  And An02g08030 | GCAGACGTTGAACGTGTAGG |

| (a) |
| --- |
| 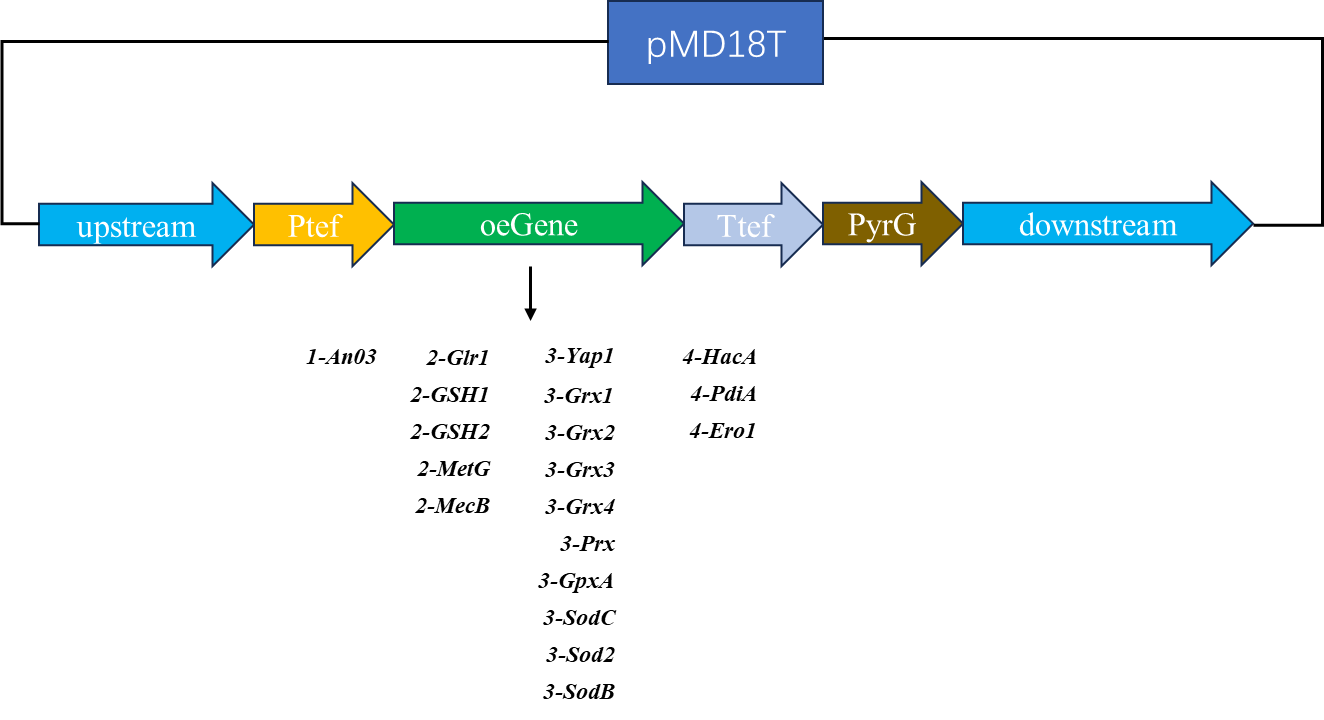 |
| (b) |
| 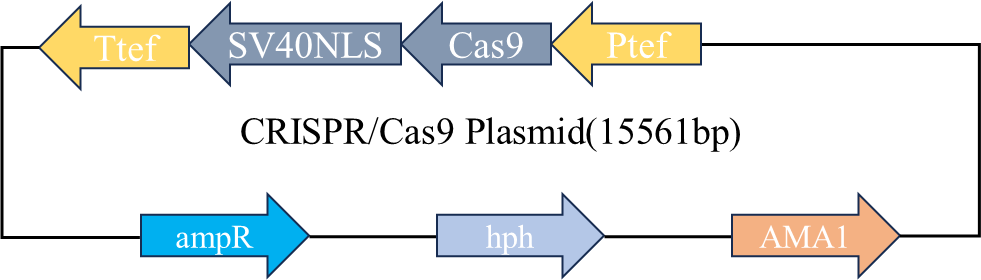  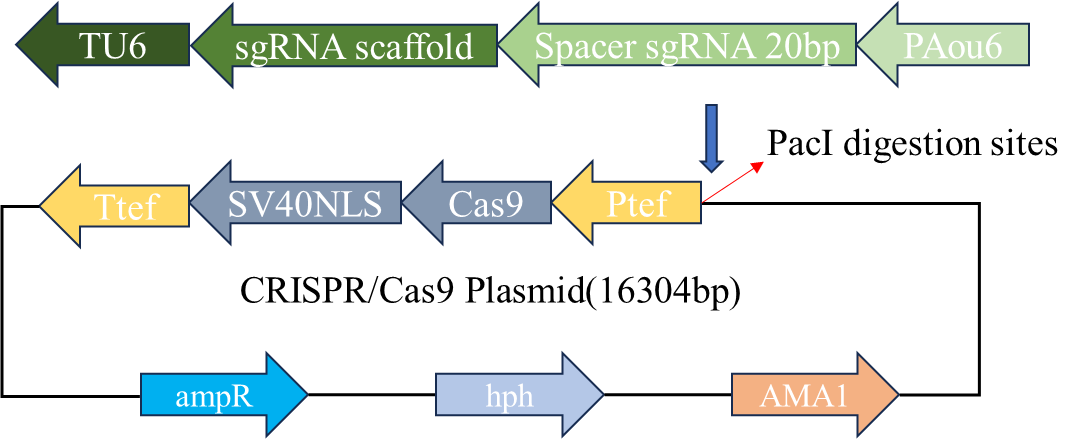 |

**Figure S1** The plasmids for expression cassettes and CRISPR/Cas9 utilized in this study.(**A**) The plasmids for expression cassettes utilized in this study.(**B**) The CRISPR/Cas9 plasmid utilized in this study.

| (a) |
| --- |
| 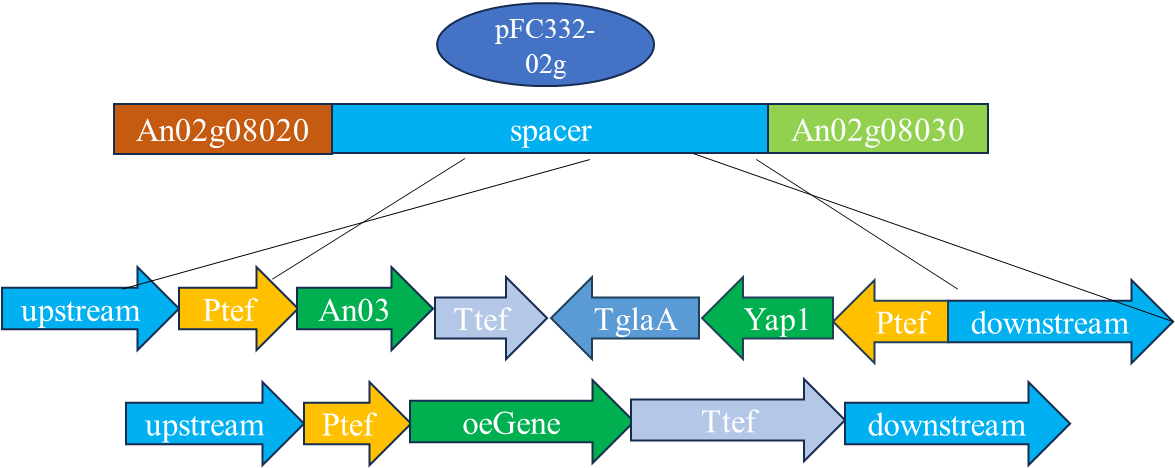 |
| (b) |
| 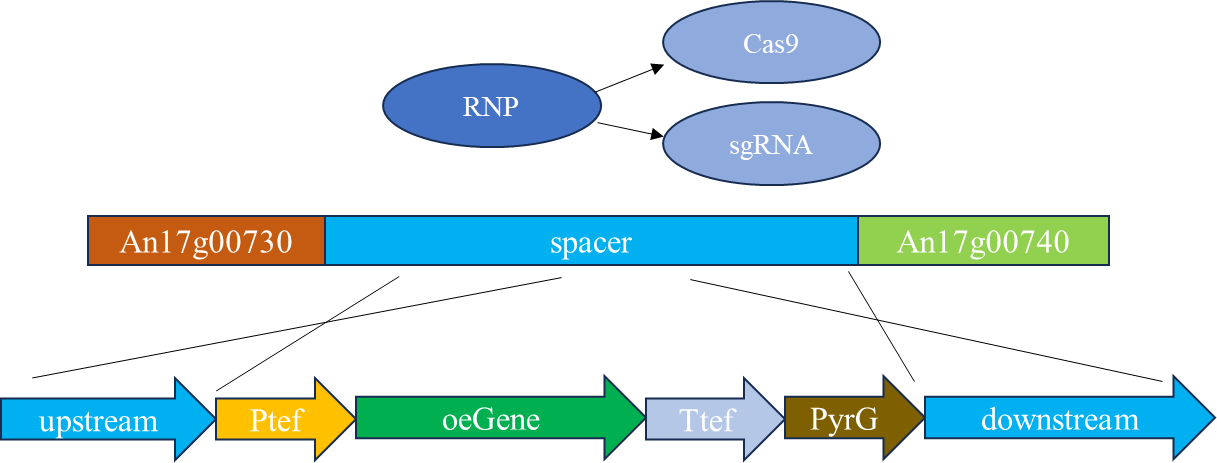 |

**Figure S2** Schematic representation of gene editing using CRISPR/Cas9 plasmid and RNP complexes.(**A**) Gene editing was performed using the CRISPR/Cas9 plasmid, where the molar ratio of CRISPR/Cas9 plasmid to repair template was 1:10.(**B**) Gene editing using RNP complexes in which the total amount of repair template is 50-100ug.


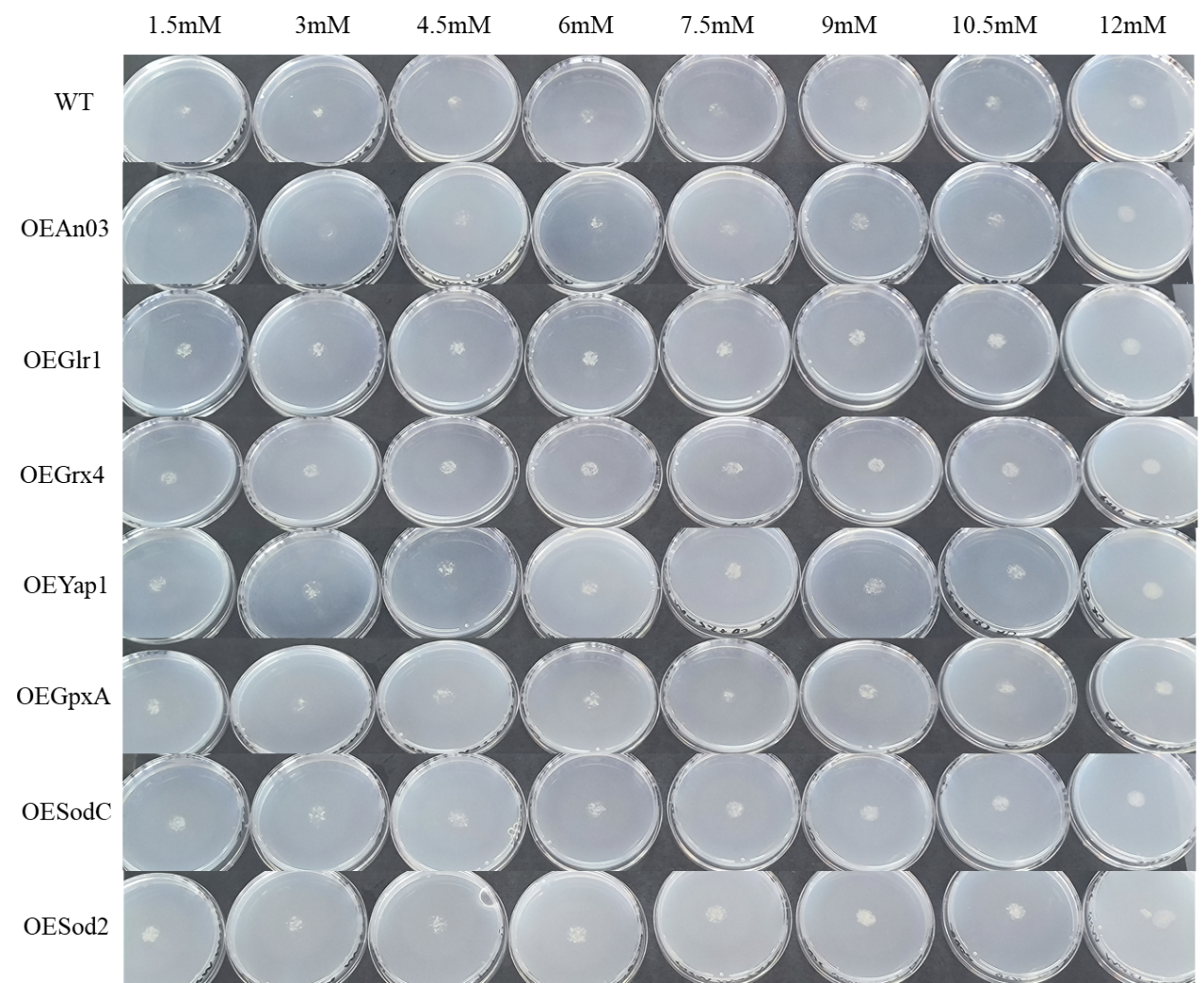


**Figure S3** presents the growth patterns of both control and mutant strains on agar plates subjected to incremental concentrations of MSB, ranging from 1.5 mM to 12 mM.

| (A) |
| --- |
| 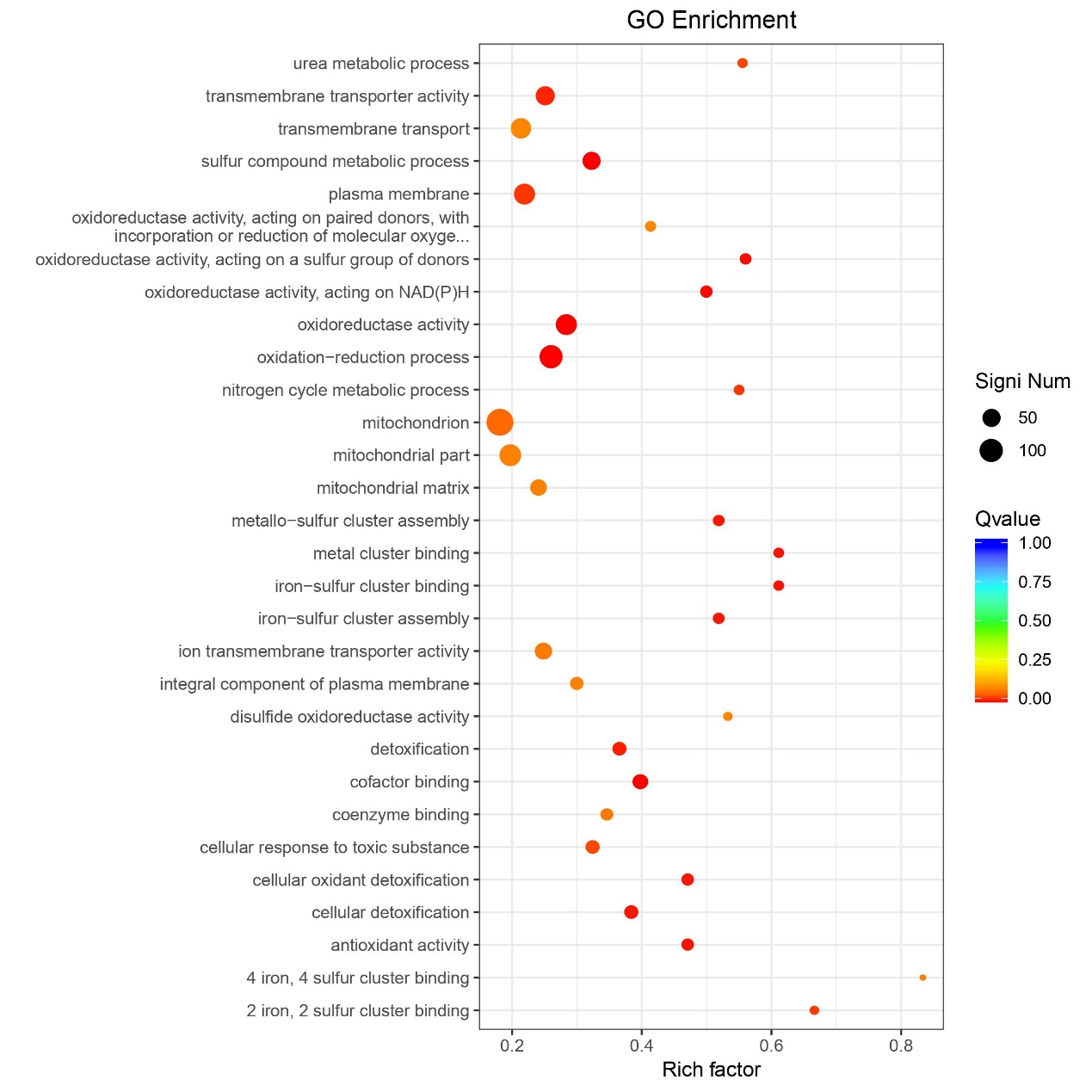 |

**Figure S4** Significantly enriched GO scatter plots for OEAn03 and WT **(A)**shows the GO enrichment in OEAn03, with the vertical axis denoting function annotation information and the horizontal axis representing the Rich factor. The Rich factor is determined by dividing the number of differentially expressed genes ascribed to a certain function by the total number of genes annotated to that function. The color intensity of each dot is proportional to the Q-value magnitude; a lower Q-value produces a color closer to red. Furthermore, the dot size represents the number of differentially expressed genes for each function. To ensure clarity, only the top 30 Gene Ontology (GO) terms with the highest enrichment degree were plotted.
